# 1T1R Nonvolatile Memory with Al/TiO_2_/Au and Sol-Gel-Processed Insulator for Barium Zirconate Nickelate Gate in Pentacene Thin Film Transistor

**DOI:** 10.3390/ma10121408

**Published:** 2017-12-09

**Authors:** Ke-Jing Lee, Yu-Chi Chang, Cheng-Jung Lee, Li-Wen Wang, Yeong-Her Wang

**Affiliations:** Institute of Microelectronics, Department of Electrical Engineering, National Cheng-Kung University, Tainan 701, Taiwan; hugh_2224@hotmail.com (K.-J.L.); s0964348@hotmail.com (Y.-C.C.); s.w.l.f.dd@gmail.com (C.-J.L.); jk220052@gmail.com (L.-W.W.)

**Keywords:** one transistor and one resistor (1T1R), organic thin-film transistor (OTFT), resistive random access memory (RRAM), sol-gel

## Abstract

A one-transistor and one-resistor (1T1R) architecture with a resistive random access memory (RRAM) cell connected to an organic thin-film transistor (OTFT) device is successfully demonstrated to avoid the cross-talk issues of only one RRAM cell. The OTFT device, which uses barium zirconate nickelate (BZN) as a dielectric layer, exhibits favorable electrical properties, such as a high field-effect mobility of 2.5 cm^2^/Vs, low threshold voltage of −2.8 V, and low leakage current of 10^−12^ A, for a driver in the 1T1R operation scheme. The 1T1R architecture with a TiO_2_-based RRAM cell connected with a BZN OTFT device indicates a low operation current (10 μA) and reliable data retention (over ten years). This favorable performance of the 1T1R device can be attributed to the additional barrier heights introduced by using Ni (II) acetylacetone as a substitute for acetylacetone, and the relatively low leakage current of a BZN dielectric layer. The proposed 1T1R device with low leakage current OTFT and excellent uniform resistance distribution of RRAM exhibits a good potential for use in practical low-power electronic applications.

## 1. Introduction

Resistive random access memory (RRAM) has attracted considerable attention due to its advantages of high-density integration, high switching speed, low power consumption, extended retention time, high operational speed, and non-destructive readout. However, a cross-point array built with only a RRAM cell suffers from unavoidable cross-talk interference because of its leakage current paths, which pass through neighboring unselected cells with low resistance. To solve this issue, another functional device should be combined with the memory device. Recently, Wan et al. [1] reported that the cross-talk issue could be reduced by an internal transistor. Organic thin-film transistors (OTFTs) have been studied intensively for their potential application in integrated circuits, their lightweight roll-to-roll printing compatibility, their flexibility, and the ease of tailoring their optoelectronic properties. Pentacene has been extensively studied as a p-type semiconductor in organic transistors, due to its favorable semiconducting behavior and high hole carrier mobility [2]. In addition, a gate oxide material with a high-κ is urgently required to achieve high mobility and low operation voltage OTFTs. However, the disadvantage of most high-κ materials is their relatively narrow bandgap, which results in aggravated gate leakage current. Owing to the leakage current of the transistor caused by a high resistive state (HRS) current of the 1T1R RRAM device, a suitable high-κ material that satisfies the sufficient wide bandgap and relatively low leakage current of the transistor is necessary to reduce the power consumption of the 1T1R RRAM device. The present study proposes a high-κ barium zirconate nickelate (BZN) dielectric layer for OTFT applications [3]. The gate leakage of the BZN-based OTFTs is reduced compared with barium zirconate oxide (BZO). This result may be attributed to adding Ni (II) acetylacetone in BZO, which increases the barrier height and reduces the leakage current. Thus, the 1T1R architecture with low leakage current BZN-based OTFT device connected to a RRAM cell is designed to reduce the power consumption and avoid cross-talk issues present when using only one RRAM cell. In addition, several 1T1R devices still require a forming process, although the cross-talk issue can be reduced by the internal transistor [4,5]. A high-voltage forming process is avoided in the 1T1R device, as a result of the sufficient oxygen vacancies possessed by the TiO_2_ thin film. The 1T1R RRAM devices exhibit low HRS currents, low operation currents, low power consumption, and reliable data retention.

## 2. Results

**Figure 1:**
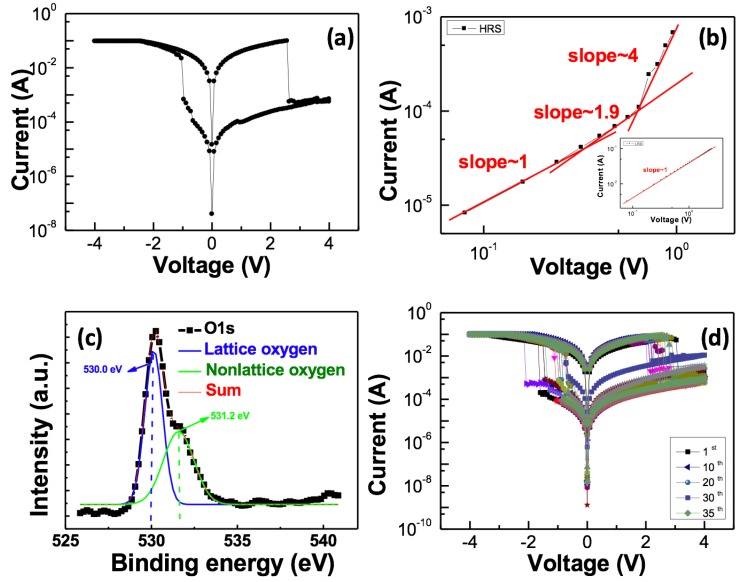
(**a**) *I–V* curves of the TiO_2_ RRAM (resistive random access memory) device; (**b**) *logI*–*logV* characteristics for the switching mechanisms; (**c**) Displays the normalized XPS (X-ray photoelectron spectroscopy) spectra for O1s of the TiO_2_ thin film; (**d**) Switching behavior of the TiO_2_ RRAM device after 35 DC (direct current) sweep cycles.

Figure 1a illustrates the switching *I*–*V* curves of the Al/TiO_2_/Au structure under the direct current (DC) voltage sweep. The voltage was applied to the top Al electrode in a sequence of +4 V → 0 V → −4 V → 0 V → +4 V. Figure 1b depicts the logarithmic plots of the *I*–*V* curves for the positive and negative voltage sweep regions, which were created to investigate the switching mechanism of the TiO_2_-based RRAM. The slope is unequal to 1, although the *ln*(*I*)–*V*^1/2^ and *ln*(*I/V*)–*V*^1/2^ plots of the *I*–*V* curves at the HRS were also investigated.

In the low-voltage region of the HRS, the current was linearly proportional to the applied bias and showed a typical Ohmic behavior. In the high-voltage region of the HRS, the *I*–*V* slopes of the fitting line were approximately 2. The current density abruptly increases with *I*–*V* slopes greater than 2 when all the available traps are filled [6,7]. X-ray photoelectron spectroscopy (XPS) was used to analyze the TiO_2_ thin films as presented in Figure 1c. The peaks located at 529.5 and 531.1 eV were associated with lattice and non-lattice oxygen ions, respectively. In the XPS results, the TiO_2_ thin film contains defects, such as non-lattice oxygen ions. Defects in the TiO_2_ thin film can form trap sites below the conduction band, where the injected charge carriers can be entrapped. Therefore, the fitting results at the HRS indicate that the space charge limited current (SCLC) model is the dominant mechanism. In the positive-voltage region, the current state maintains a low resistive state (LRS) and demonstrates an Ohmic conduction behavior with a slope of approximately 1, thereby confirming the formation of a conductive filament [8]. Figure 1d illustrates the TiO_2_-based RRAM that can maintain an on/off ratio of over 10^2^ after continuous DC voltage switching cycles of 35.

**Figure 2:**
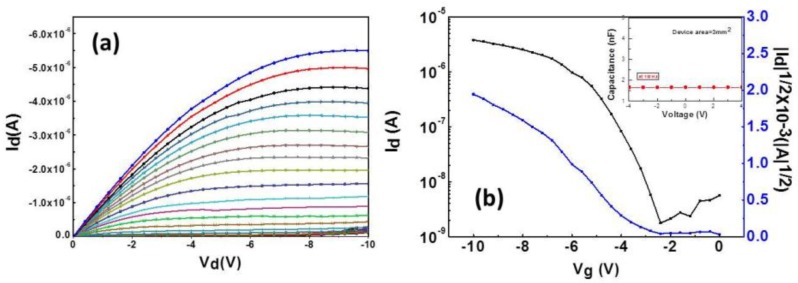
(**a**) *C–V* (current–voltage) output and (**b**) transfer characteristics of the BZN (barium zirconate nickelate)-based OTFT (organic thin film transistor) in the 1T1R device. The inset displays the *C–V* characteristic of the Au/BZN/Al capacitor. The frequency in the *C–V* measurement is set to 1 MHz.

**Figure 3:**
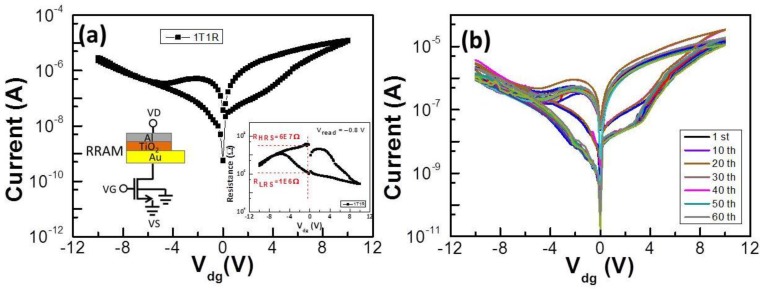
(**a**) *I–V* characteristics and schematics of the 1T1R (one-transistor and one-resistor) device. The inset shows the *R–V* (resistance-voltage) curve corresponding to Figure 3a; (**b**) Switching behavior of the 1T1R device cycles after 60 DC sweep cycles.

The output and transfer characteristics of the BZN-based OTFT in the 1T1R device measured at *V*_dg_ = −10 V are depicted in Figure 2a,b. In terms of the transfer characteristics of the devices, subthreshold slope swing (S.S.), the on/off current ratio threshold, voltage (V_th_), and high field-effect mobility were approximately 1 V/decade, 10^3^, −2.8 V, and 2.5 cm^2^ V^−1^ S^−1^, respectively. The inset of Figure 2b shows the current-voltage (*C–V*) characteristic of the Au/BZN/Al capacitor. The frequency in the *C–V* measurement is set to 1 MHz. The capacitance of the BZN layers was 1.5 nF for a device area of 3 mm^2^. The TiO_2_-based RRAM cell connected to the solution BZN OTFT device was demonstrated to successfully fabricate for the 1T1R architecture. The merging of the Au/pentacene/BZN/Al transistor device with Al/TiO_2_/Au memory device as shown in the inset of Figure 3a resulted in a superimposed *I*–*V* characteristics, as illustrated in Figure 3a. As the results show, the 1T1R structure somehow presents analog behavior, while RRAM (Figure 1a) shows digital behavior, with an obvious On/Off ratio. This might be due to the effect of the pentacene transistor, and further study is needed. Although the shapes of Figure 1a and Figure 3a look somehow different, the current levels are all effectively suppressed by two orders of magnitude under the 1T1R structure. In the set process, a positive voltage is applied on the top electrode sweeping from 0 to a set voltage of 3.8 V, and a set current of 0.23 μA is obtained with the source grounded. This process causes the current to suddenly increase to reach LRS, where the value of the set current is controlled by modulating the gate voltage of the transistor. The inset of Figure 3a shows the resistance resistance-voltage (*R–V*) curve corresponding to Figure 3a. A read voltage of −0.8 V indicates a resistance of 60 MΩ for the HRS and 1 MΩ for the LRS, corresponding to a ratio of 60.

Figure 3b demonstrates the 1T1R memory device without obvious deterioration after 60 successive DC resistive switching cycles. The shapes of the *I*–*V* curves and the first sweep were similar, and only minimal voltage and resistance variations were observed, thereby indicating an excellent stability in its bipolar resistive switching behavior. The memory cell can be switched to the on-state for *V*_dg_ at 10 V by applying a positive voltage *V*_dg_ at the gate of the control transistor. The cell can be switched back to the off-state for *V*_dg_ = 0 V.

**Figure 4:**
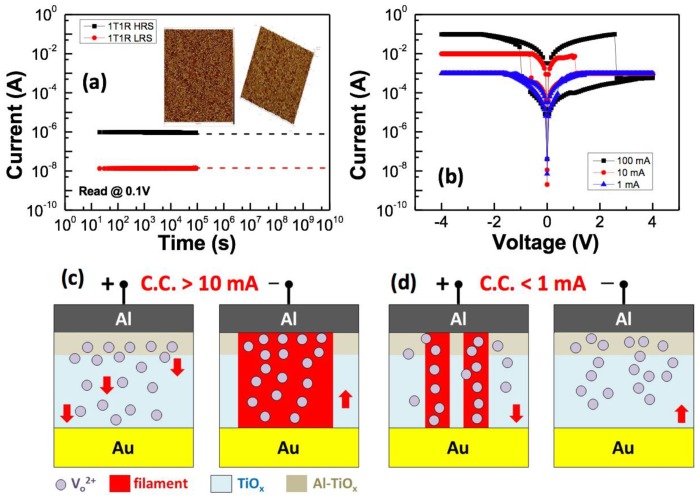
(**a**) Data retention results of HRS (high resistive state) and LRS (low resistive state) for Al/TiO_2_/Au/pentacene/BZN/Al 1T1R devices at RT (room temperature) obtained at 0.1 V. The inset image displays the AFM (atomic force microscopy) topography image of the TiO_2_ thin film. (**b**) Dependence of the *I*–*V* curves on the TiO_2_ RRAM device with the different compliance currents. The schematic of the proposed switching mechanism indicates that the compliance current of (**c**) >10 mA and (**d**) <1 mA was applied to the Al/TiO_2_/Au RRAM device.

Figure 4a presents the retention capabilities of the Al/TiO_2_/Au/pentacene/BZN/Al 1T1R devices measured at room temperature (RT) obtained at a voltage of 0.1 V. For the Al/TiO_2_/Au/pentacene/BZN/Al 1T1R device, a 10-year on/off ratio of over 10^2^ can be extrapolated at RT. The inset in Figure 4a illustrates the surface topography of the TiO_2_ surface. The root-mean-square roughness (R_rms_) value of the TiO_2_ thin film is approximately 1.3 nm. The smooth roughness facilitates the stable resistive switching of the device [9]. Thus, the favorable retention capability can be partly attributed to the smooth TiO_2_ thin film.

The RRAM device exhibits bipolar behavior (the set process was triggered by a negative voltage, while the reset process occurred under a positive bias) when the compliance current is more than 10 mA. The device performs a reversed switching polarity when using a low compliance current of 1 mA, as displayed in Figure 4b. Similarly, the 1T1R device can be turned on/off by applying a positive/negative voltage, respectively. The switching current of the 1T1R device is lower than 10 µA. The distinct switching is attributed to different compliance currents. This phenomenon is due to the large oxygen vacancy in the Al-TiO_x_ layer, and the resulting asymmetric oxygen vacancy profile.

The redox reactions of active electrodes at the electrode/insulator interface are considered to have an important role in the resistive switching behavior [10]. The spontaneous oxidation of Al at the Al-TiO_2_ interface causes the formation of AlO_x_, because Al is chemically active. The interface AlO_x_ layer acts as a diffusion barrier that prevents oxygen from infusing into the atmosphere, and stores and releases oxygen during switching, similar to an oxygen reservoir [11,12].

Figure 4c,d depicts the schematics of the proposed switching mechanism. An oxygen-deficient layer was preferentially produced naturally in the TiO_x_ layer near the Al electrode when the compliance current was more than 10 mA. Moreover, a certain fraction of the Al in the top electrode was diffused into the TiO_x_ layer. The oxygen-deficient layer can be considered an Al-TiO_x_ layer. Al^3+^ substitutes for Ti^4+^ within the TiO_x_ for the Al-TiO_x_ layer, and oxygen vacancies can be produced by diffused Al^3+^. Thus, more oxygen vacancies were observed in the Al-TiO_x_ layer than in the TiO_x_ layer. Oxygen vacancies migrate toward the Al top electrode when a negative voltage is applied to the Al top electrode and form the filament in layers after a sufficient electric field is reached, thereby bringing the device into the LRS. By contrast, oxygen vacancies from the Al-TiO_x_ layer move toward the Au bottom electrode when a positive voltage is applied to the Al top electrode, thus causing the filament to rupture, placing the device into the HRS. The HRS is difficult to reach during normal device operations when the compliance current is less than 1 mA. The thin film behaves similarly to a conductor, because the compliance current or positive bias is not high enough to sufficiently deplete the oxygen vacancy in the Al-TiO_x_ layer. Thus, the polarity of bipolar switching is found to be determined by the compliance current with different distributions of the internal oxygen vacancies used in the RRAM and 1T1R devices.

## 3. Materials and Methods

The OTFT was fabricated onto a Corning glass Eagle 2000 substrate. First, the glass substrate was cleaned with acetone, ethanol, and de-ionized water. An approximately 38 nm-thick Aluminum (Al) was deposited as the gate electrode via a radio-frequency (RF) magnetron sputtering system.

**Figure 5:**
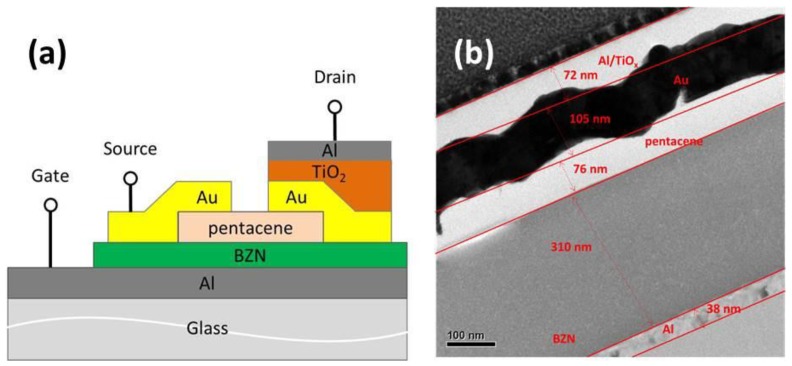
(**a**) Cross-sectional view of the 1T1R memory device. (**b**) Corresponding TEM (transmission electron microscopy) image of the Al/TiO_2_/Au/pentacene/BZN/Al/glass stacked structure.

The barium acetate, zirconium *n*-propoxide, and nickel II acetylacetone were synthesized by using the various sol-gel techniques. The required amount of the barium acetate (536.8 mg) was dissolved in glacial acetic acid (5 mL) by stirring, and then was heated on a 120 °C hot plate until complete dissolution was achieved (A1 solution). The nickel II acetylacetone (543.7 mg) and zirconium *n*-propoxide (65 μL) were mixed with acetylacetone (470 μL) and dissolved in 2-methoxyethanol (3 mL) by stirring (A2+B solution). Subsequently, A1 was slowly dropped into A2+B until a completely transparent solution was obtained. 2-methoxyethanol (1.5 mL) was added to acquire a 0.5 M BZN sol precursor. The BZN solution was spin-coated onto the Al/glass substrate with a thickness of 310 nm as the gate dielectric after the solution was completely prepared using the sol-gel process. The BZN layer was baked at 100 °C for 10 min under ambient air conditions. The pentacene thin film (76 nm) was thermally evaporated on the BZN layer at a deposition rate of 0.08 to 0.15 nm/s. Finally, a gold (Au) electrode was deposited using the RF magnetron sputtering system and defined through shadow masks as the source and drain. The channel length and width of the transistors were 150 and 1500 μm, respectively. For manufacturing the 1T1R configuration, the RRAM device was fabricated directly on the drain electrodes of the TFT device. A simple metal/insulator/metal structure, which consists of Al/TiO_2_/gold (Au) structures, was fabricated. First, Au was deposited to be a bottom electrode of the RRAM device through the RF magnetron sputtering system. Then, the 17 nm TiO_2_ layer was deposited onto the Au electrode by sputtering. Finally, a 68 nm-thick Al was deposited as the top electrode. The devices were measured at RT (300 K) with the humidity conditions of 40–50%. All measured data were extracted from the devices with an area of 3 mm^2^, as demonstrated by the diagram of the Al/TiO_2_/Au/pentacene/BZN/Al/glass in Figure 5. The electrical properties of the transistors were analyzed using an Agilent B1500 semiconductor parameter analyzer.

## 4. Conclusions

The 1T1R architecture with a RRAM cell connected to the solution BZN OTFT device was successfully demonstrated. The TiO_2_-based RRAM device with bipolar mode shows an on/off ratio of 10^2^ and a uniform resistance distribution. The favorable performance of the 1T1R device can be attributed to the low leakage current of the BZN-based OTFT. The 1T1R devices exhibit a low operation current of 10 μA and a reliable data retention of over ten years, because the TiO_2_-based RRAM and BZN-based OTFT demonstrate a stable device yield and favorable device performance. The low power consumption and excellent uniform resistance distribution of the proposed 1T1R RRAM device indicate a considerable potential for practical applications.
